# Anticardal Operation for Aneurism

**Published:** 1830

**Authors:** 


### II. Analytical.

20. *Anticardial operation for aneurism.*—A peculiar *felicity* appears to attend the surgical operations of our distinguished countryman, Dr. Mott. In performing those formidable operations, which the most intrepid and skilful surgeons have attempted only with a faint hope of pointing out a remote prospect of success, or of directing others with greater certainty through the difficulties that surround them, his success has exceeded the most sanguine expectations of the profession.

A short historical notice of "Brasdor's operation," was given in the No. of this Journal for October 1829; and we are now gratified to inform our readers that it has been successfully performed in this country. From the account of the operation, published by Dr. Mott in the American Journal of the Medical Sciences, we make the following extracts.

Moses R. Gardner, ætat. 51, by profession a farmer, of sound constitution and good habits of life, applied to me some time in March for advice.

He gave the following relation of his case:—About three years ago, while occupied in removing a building, and compelled to lift heavy weights, he was attacked with pain in the upper and back part of the neck. This lasted until the month of January, when it extended to the right shoulder and arm, and continued until the following May; it then partially subsided, and he observed his voice was becoming hoarse, which he attributed to exposure and consequent cold. About eighteen months since, while shaving, he discovered a small swelling at the upper part of the breast bone, but did not remark any throbbing in it until some time afterwards. He had consulted a physician, but received no positive opinion on the case.

Upon examination, I found above the sternum a pulsating tumour, about the size of a pigeon's egg, spreading some distance under the clavicular and sternal portions of the right sterno-mastoideus muscle, in the course of the subclavian artery, and extending as low down upon the pleura as the second rib, compressing more or less the bronchial tubes, and producing on the least coughing or exercise a wheezing, not unlike that of asthma. He shrunk from the least pressure upon it; complaining of impeded respiration, followed by pain. Its pulsations were synchronous with those of the heart, and decidedly aneurismal.

Having tried a palliative course without benefit, he returned to the city on the 12th of September.

I found the tumour above the sternum had increased to the size of a large walnut, and upon a careful application of the stethoscope, it was evidently encroaching more upon the chest. The whizzing sound, (*bruit de soufflet,*) could be heard; thoracic viscera were sound, the respiratory murmur being distinct throughout. His respiration was very much impeded by speaking, walking, or coughing, and almost entirely suspended by the least pressure upon the tumour: the action of the right carotid was much more feeble than that of the left; no pulsation could be discovered in its branches; the right subclavian, external to the scaleni muscles, was natural, while the axillary and brachial arteries could hardly be felt; at the wrist no pulse could be found; the pulsations of the arteries of the left side were natural. His general health was good.

In reflecting upon this case, and comparing the relative situation of the parts, I was persuaded the aneurism was of the arteria innominata, involving the subclavian and the root of the carotid; having formed this conclusion, I considered it a proper case for the operation proposed by Brasdor, and recently so ably revived, and first successfully performed by the distinguished Wardrop, whose scientific researches and masterly views of this subject have since been so fully confirmed by himself and others.

I thought further delay unnecessary, and he being willing to abide by my judgment after having stated to him the chances of the operation, I resolved on its performance. From the evident interruption in the circulation of the right arm, and the apparent effort of nature to effect a spontaneous cure, I determined upon tying the carotid first, to observe the result, and afterwards to secure the subclavian, should it be required.

On the 26th of September I operated. The artery was taken up in the usual manner; no material change was observed.

The patient was kept for some time on a low diet; an occasional purgative was prescribed; and, as he had been accustomed to opiates, a tea spoonful of laudanum was permitted for a few nights. The unpleasant symptoms were immediately mitigated; the pulsation, soreness and tenseness of the tumour diminished; the voice became more natural, and the patient could be more in the recumbent position. On the 28th, the pulsation in the radial artery became very distinct, intermitting once in 10 or 15 beats; but it again became indistinct and gradually disappeared; and at the same time the arm became œdematous, and the patient complained of inability to use it. The ligature separated on the 16th, by which time the tumour and pulsation had disappeared.

22*d.*—Wound just healed; weakness of the arm very considerable; fingers very thick and clumsy; arm swelled and pits upon pressure; no pulse in the right radial artery: breathing very easy: cough and expectoration much less; can sleep easy in any position, which he has not been able to do for many months.

26*th.*—Left town this morning for his residence in New Jersey.

